# 

**Published:** 1896-07-25

**Authors:** 


					XXImiinwuriTAiJi ABSOLUTELY PURE, therefore BESTT" July 25th, 1896.
f? CADBURY'S ^ m ^ CADBURY'S
(y LBEEDo' cocoa JPtJ cocoa
*' The standard of highest purity at present llL V ?
B Lattajnable."?Lancet, ALKALIES USED(
mm
Jm ?*
c
ORWUH HOSPI.TAJ. "
"I inni rurilT T?T?.Tf?T! TVAPCW/IT!
?UiiAdUUrr fFttf I CttV* IDULMMROM
No. 513.\Syo1. XX.
^Saturday,
July 25th, 1896.
WITH EXTRA NURSING SUPPLEMENT. *?HL3KKS5S"-
[Registered at the General Post Offloe as a Newspaper for Monthly Parts, 6d.; post free 83
transmission in the United Kingdom and Abroad.] Ditto per Annum, 8s. 6d., post free".

				

